# Proliferative memory SAMHD1^low^ CD4^+^ T cells harbour high levels of HIV-1 with compartmentalized viral populations

**DOI:** 10.1371/journal.ppat.1007868

**Published:** 2019-06-20

**Authors:** Lylia Hani, Antoine Chaillon, Marie-Laure Nere, Nicolas Ruffin, Joudy Alameddine, Maud Salmona, José-Luiz Lopez Zaragoza, Davey M. Smith, Olivier Schwartz, Jean-Daniel Lelièvre, Constance Delaugerre, Yves Lévy, Nabila Seddiki

**Affiliations:** 1 Inserm, U955 Equipe 16, Créteil, France; 2 Université Paris Est, Faculté de médecine, Créteil, France; 3 Vaccine Research Institute (VRI), Créteil, France; 4 Department of Medicine, University of California San Diego, CA, United States of America; 5 Hôpital Saint Louis, INSERM U944, Université de Paris, Paris, France; 6 AP-HP, Hôpital H. Mondor—A. Chenevier, Service d'immunologie clinique et maladies infectieuses, Créteil, France; 7 Unité Virus et Immunité, Département de Virologie, Institut Pasteur, Paris, France; Emory University, UNITED STATES

## Abstract

We previously reported the presence of memory CD4^+^ T cells that express low levels of SAMHD1 (SAMHD1^low^) in peripheral blood and lymph nodes from both HIV-1 infected and uninfected individuals. These cells are enriched in Th17 and Tfh subsets, two populations known to be preferentially targeted by HIV-1. Here we investigated whether SAMHD1^low^ CD4^+^ T-cells harbour replication-competent virus and compartimentalized HIV-1 genomes. We sorted memory CD4^+^CD45RO^+^SAMHD1^low^, CD4^+^CD45RO^+^SAMHD1^+^ and naive CD4^+^CD45RO^-^SAMHD1^+^ cells from HIV-1-infected patients on anti-retroviral therapy (c-ART) and performed HIV-1 DNA quantification, ultra-deep-sequencing of partial *env* (C2/V3) sequences and phenotypic characterization of the cells. We show that SAMHD1^low^ cells include novel Th17 CCR6^+^ subsets that lack CXCR3 and CCR4 (CCR6^+^DN). There is a decrease of the % of Th17 in SAMHD1^low^ compartment in infected compared to uninfected individuals (41% vs 55%, p<0.05), whereas the % of CCR6^+^DN increases (7.95% vs 3.8%, p<0.05). Moreover, in HIV-1 infected patients, memory SAMHD1^low^ cells harbour high levels of HIV-1 DNA compared to memory SAMHD1^+^ cells (4.5 vs 3.8 log/10^6^cells, respectively, p<0.001), while naïve SAMHD1^+^ showed significantly lower levels (3.1 log/10^6^cells, p<0.0001). Importantly, we show that SAMHD1^low^ cells contain p24-producing cells. Moreover, phylogenetic analyses revealed well-segregated HIV-1 DNA populations with compartmentalization between SAMHD1^low^ and SAMHD1^+^ memory cells, and limited viral exchange. As expected, the % of Ki67^+^ cells was significantly higher in SAMHD1^low^ compared to SAMHD1^+^ cells. There was positive association between levels of HIV-1 DNA and Ki67^+^ in memory SAMHD1^low^ cells, but not in memory and naïve SAMHD1^+^ CD4^+^ T-cells. Altogether, these data suggest that proliferative memory SAMHD1^low^ cells contribute to viral persistence.

## Author summary

In our previous results we reported that memory CD4^+^ T cells expressing low levels of SAMHD1 (SAMHD1^low^) are present in peripheral blood and lymph nodes from HIV-1 infected and uninfected individuals. These cells were enriched in Th17 and Tfh, two populations targeted by HIV-1. Here we used purified memory CD4^+^CD45RO^+^SAMHD1^low^, CD4^+^CD45RO^+^SAMHD1^+^ and naive CD4^+^CD45RO^-^SAMHD1^+^ cells from HIV-1-infected and treated patients to perform cell-associated HIV-1 DNA quantification, p24-producing cells detection, ultra-deep-sequencing of partial *env* (C2/V3) HIV-1 DNA and further phenotypic characterization. Our results demonstrate that (i) Th17 and CCR6^+^DN-expressing transcriptional signature of early Th17, two major populations that are susceptible to HIV-1 infection, are present in SAMHD1^low^ cells, and while the former decreased significantly in c-ART HIV-1 infected compared to uninfected individuals, the latter significantly increased; (ii) memory SAMHD1^low^ cells from c-ART patients carry high levels of HIV-1 DNA compared to SAMHD1^+^ cells, and these levels positively and significantly correlated with Ki67 expression; (iii) memory SAMHD1^low^ cells from patients harbour p24-producing cells; (iv) phylogenetic analyses revealed well-segregated HIV-1 DNA populations with significant compartmentalization between SAMHD1^low^ and SAMHD1^+^ cells and limited viral exchange. Our data demonstrate that memory SAMHD1^low^ cells contribute to HIV-1 persistence.

## Introduction

The remarkable stability of the latent HIV-1 reservoir in the CD4^+^ memory T cell population prevents viral eradication with current antiretroviral therapy (c-ART). HIV-1 persistence is established through two major mechanisms: constitution of a small pool of lifelong latently infected CD4^+^ T-cells early in infection, ensuring the persistence of the virus for decades during c-ART [1,2] and residual replication of the virus at low levels in anatomical reservoirs in which c-ART may not diffuse [3]. Identifying cellular markers expressed at the surface of these cells may lead to novel therapeutic strategies to eliminate, or at least reduce the size of the HIV-1 reservoir [4].

HIV-1 populations can vary in resistance to drugs, diversity and cellular tropism [5–7]. Viral compartmentalization between cellular subsets has been reported [8–10], and the dynamics of HIV-1 migration between anatomical compartments has been largely investigated [11–15]. However, the compartmentalization of HIV-1 within the same pool of activated or memory CD4^+^ T cells within the same infected individual has not been investigated.

HIV-1 can persist in several subsets of CD4^+^ with different functional properties and trafficking potential [2,16–20]. Central memory (TCM) and transitional memory (TTM) CD4^+^ T cells were identified as the major cellular reservoirs for HIV-1 during c-ART [2]. A less differentiated subset of long-lived cells with high self-renewal capacity, the stem-cell memory CD4^+^ T cells (TSCM), has been identified as a main contributor to long-term HIV-1 persistence [19,20]. Moreover, T follicular helper (Tfh) cells present in secondary lymphoid organs represent a target for HIV-1 infection and a major reservoir during c-ART [21,22]. T regulatory (Tregs), Th17, Th1/Th17 CD4^+^ T cells, in addition to cells co-expressing CCR6 and CXCR3, show increasing contribution to the viral reservoir during c-ART [16,23–25]. Recently, Wacleche et al. identified two novel Th17-polarized subsets, lacking (double negative, CCR6^+^DN) or co-expressing CXCR3 and CCR4 (double positive, CCR6^+^DP) that support a major contribution of HIV-1 persistence during c-ART [18]. Further evidence focusing on elucidating the mechanism(s) of viral dynamics and persistence in T-cell subsets are still needed.

SAMHD1, a dNTP triphosphohydrolase, restricts productive HIV-1 infection in dendritic cells, myeloid cells [26,27] and resting CD4^+^ T cells [28,29]. SAMHD1, through its deoxynucleoside triphosphates (dNTPs) hydrolase function, controls the intracellular dNTP pool [28–30]. The restriction of HIV-1 infection by SAMHD1 occurs via its ribonuclease activity and phosphorylation of SAMHD1 inhibits this restrictive function [31–33].

Recently, we reported that circulating memory CD4^+^ T cells comprise a population of cells expressing low levels of SAMHD1 [34]. Those memory SAMHD1^low^ CD4^+^ T cells were highly differentiated and activated (CD38^+^, PD-1^+^, HLA-DR^+^), exhibited a large proportion of cycling cells and were enriched in Th17 in peripheral blood. We also demonstrated that SAMHD1^low^ cells were more susceptible to HIV-1 infection and thus preferentially depleted in HIV-1 infected individuals. In secondary lymphoid organs, Tfh cells from infected patients lacked the expression of SAMHD1, which was accompanied by a higher susceptibility to HIV-1 infection *in vitro* [34]. Hence, our previous study strongly suggested that SAMHD1^low^ cells might be a preferential target during HIV-1 pathogenesis. Yet, the fate of SAMHD1^low^ memory CD4^+^ T cells as a reservoir, *in vivo*, remains unknown.

It is well established that memory CD4^+^ T cells comprise an important HIV-1 reservoir preventing the rise of a cure. As SAMHD1^low^ cells may support HIV-1 replication *in vivo*, we aimed to determine the contribution of these cells to HIV-1 reservoir and to study viral exchange between CD4^+^ subsets to better characterize the HIV-1 reservoir formation, spread and fate, as well as to give insight in T cell differentiation *in vivo* in HIV-1 infected patients.

## Results

### Memory CD4^+^ SAMHD1^low^ cells are depleted in c-ART HIV-1 infected patients and are enriched in Th17-polarized CCR6^+^ populations

We have previously demonstrated that peripheral blood memory SAMHD1^low^ CD4^+^ T cells were enriched in CXCR3^-^CCR6^+^CCR4^+^ Th17 cells [34]. Here we sought to determine whether SAMHD1^low^ cells include novel CCR6^+^ subsets that co-express or not CXCR3 and CCR4 [35], namely double positive (DP) or negative (DN) CCR6^+^ cells. These latter cells express a unique transcriptional signature of early Th17 and have been shown to contain replication competent HIV-1 [35].

**Fig 1:**
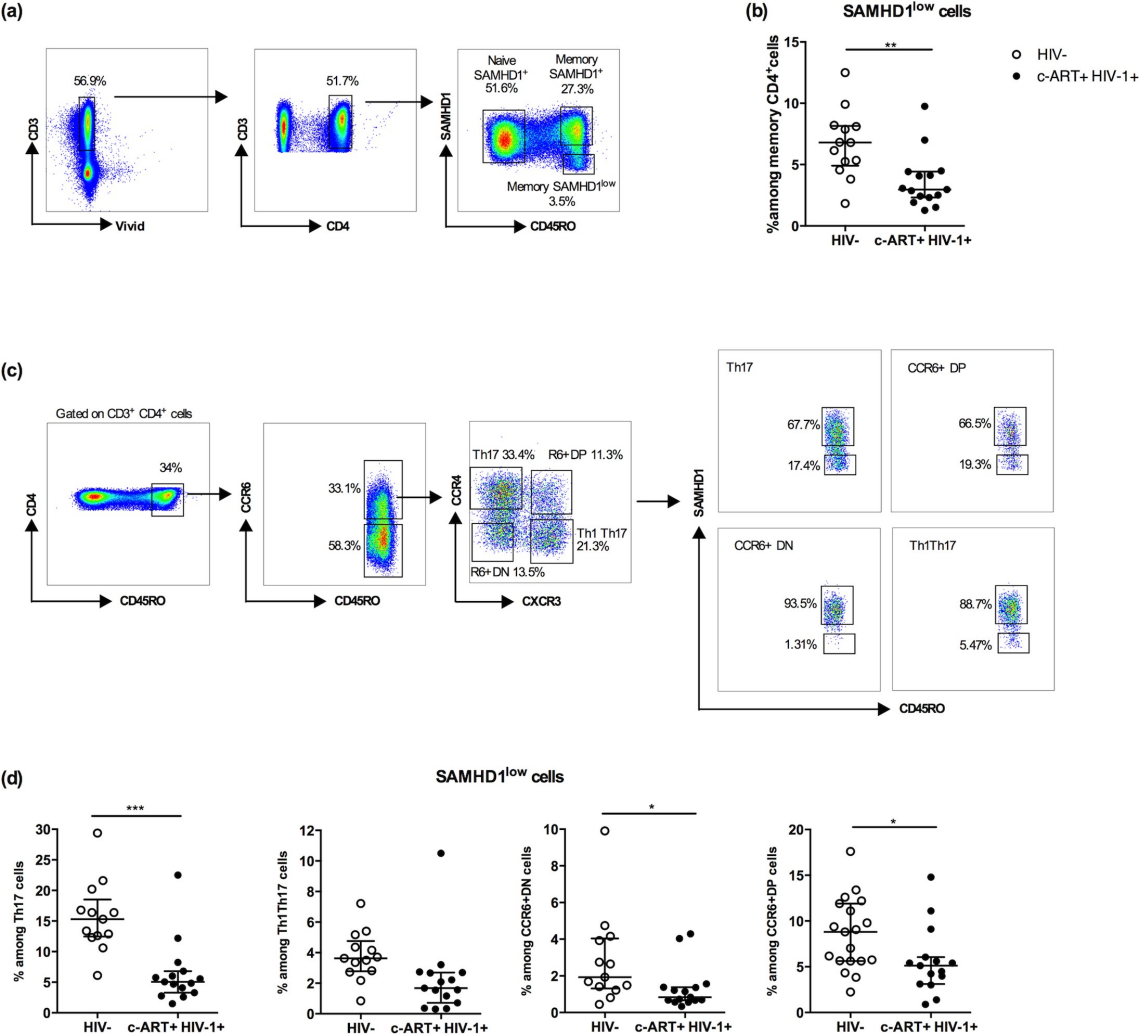
Memory CD4^+^ SAMHD1^low^ cells are depleted in c-ART HIV-1 infected patients and enriched in Th17-polarized CCR6^+^ populations. Fresh PBMCs from HIV-1 negative (HIV-, n = 13) and HIV-1 infected individuals receiving c-ART (cART+ HIV-1+, n = 17) were stained with a cocktail of fluorochrome-conjugated -CD3, -CD4, -CD45RO, -CXCR3, -CCR4, -CCR6 and -SAMHD1 monoclonal antibodies. A viability staining was used to exclude dead cells. **(a)** Gating strategy for naïve SAMHD1^+^ (CD45RO^-^ SAMHD1^+^), memory SAMHD1^+^ (CD45RO^+^SAMHD1^+^), memory SAMHD1^low^ (CD45RO^+^SAMHD1^low^) cells. **(b)** Percentages of memory SAMHD1^low^ cells among total memory CD4^+^ T-cells from HIV- and c-ART+ HIV-1+ patients. **(c)** Gating strategy for memory SAMHD1^+^ and SAMHD1^low^ cells among T helper 17 (Th17: CD45RO^+^CXCR3^-^CCR4^+^CCR6^+^), Th1/Th17 (CD45RO^+^CXCR3^+^CCR4^-^CCR6^+^), CCR6^+^ double positive DP (CCR6^+^CXCR3^+^CCR4^+^), CCR6^+^ double negative DN (CCR6^+^CXCR3^-^CCR4^-^) cells. **(d)** Percentages of CCR6^+^ Th17, Th1/17, R6^+^ (CCR6^+^) DN, R6^+^DN cells among gated memory SAMHD1^low^ CD4^+^ T-cells in uninfected vs HIV infected individuals. Data represent median with interquartile range. *p<0.05 and ***p<0.001, unpaired t-test.

We first assessed the presence of total memory CD4^+^ SAMHD1^low^ cells (gating strategy **Fig 1A**) in peripheral blood of c-ART HIV-1 infected patients (n = 17) then determined their frequencies and compared them to uninfected individuals (n = 13). We found a significant decrease of %SAMHD1^low^ cells in patients as compared to uninfected controls (2.97% [1.27–9.75] vs 6.79% [1.83–12.5] respectively, P<0.001) **(Fig 1B)**. Moreover we examined the presence of SAMHD1^low^ cells in Th17, Th1Th17, CCR6^+^DN and CCR6^+^DP cell populations using similar gating strategies as previously reported (**Fig 1C** and [18]). We found a significant decrease of %SAMHD1^low^ cells amongst Th17, CCR6^+^DN and CCR6^+^DP populations in c-ART HIV-1^+^ patients as compared to uninfected controls (5.07% vs 15.3% P<0.001, 0.84% vs 1.93% P<0.05 and 5.12% vs 8.82% P<0.05, respectively) **(Fig 1D)**, while we did not observe any significant difference in Th1Th17 compartment (1.68% vs 3.63%, p>0,05). Of note, we did not observe any difference in the characteristics of the two patients who exhibited higher %SAMHD1^low^ cells (**Fig 1B** and **1D**).

**Fig 2:**
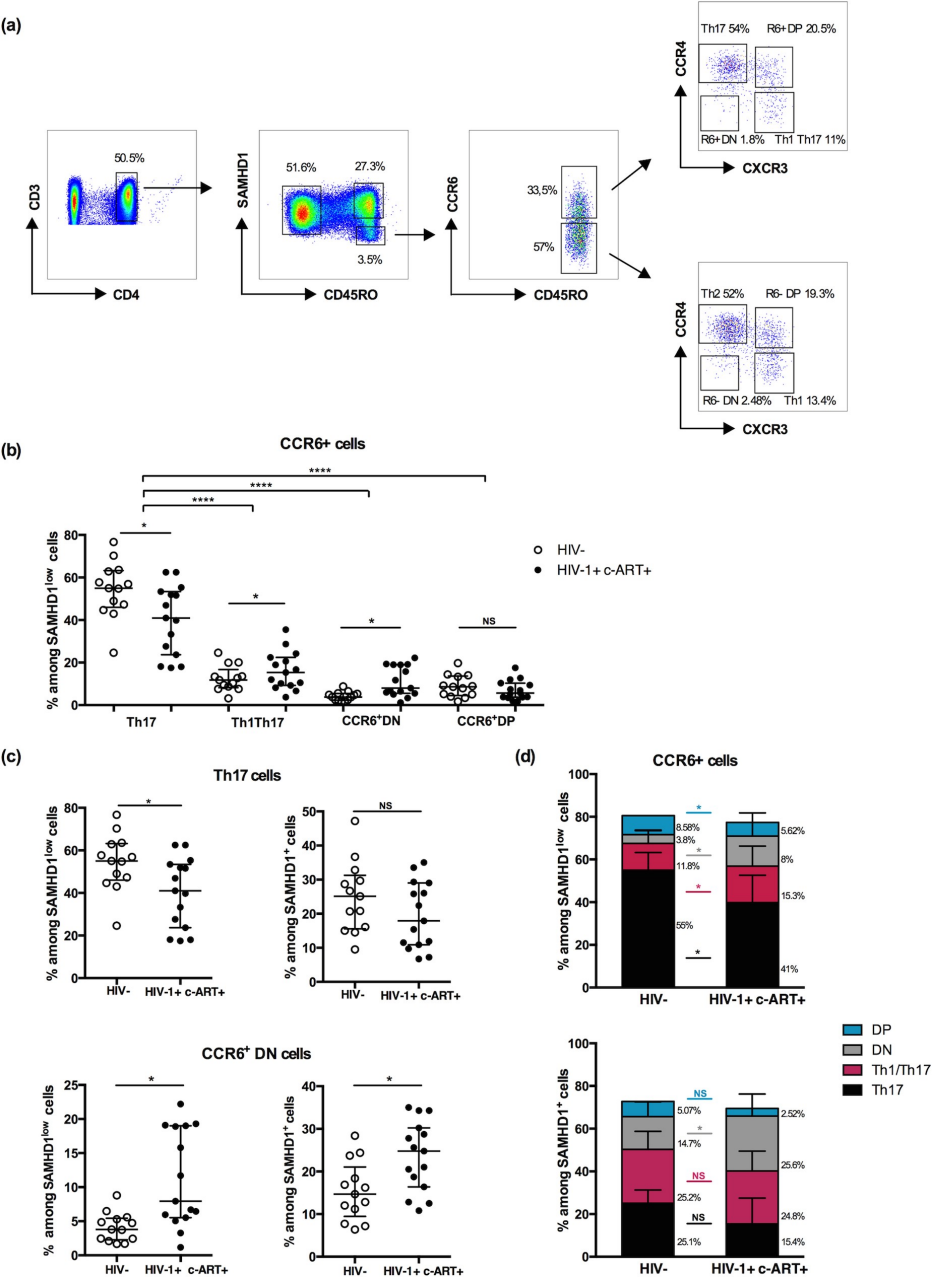
Memory CD4^+^ SAMHD1^low^ cells are enriched in Th17 cells. **(a) Gating strategy for** CCR6^+^ Th17, Th1/17, R6^+^DP and R6^+^DN cells. (**b**) Percentages of CCR6^+^ Th17, Th1/17, R6^+^DP, R6^+^DN cells among gated memory SAMHD1^low^ CD4^+^ T-cells in uninfected vs HIV infected individuals **(c)** Percentages of CCR6^+^ Th17 and R6^+^DN cells among gated memory SAMHD1^low^ and SAMHD1^+^ CD4^+^ T-cells in uninfected vs HIV infected individuals. **(d)** Percentages of CCR6^+^ Th17, Th1/17, R6^+^DP and R6^+^DN cells among gated memory SAMHD1^low^ and SAMHD1^+^ CD4^+^ T-cells. Data represent median with interquartile range. *p<0.05, t-test and 2-way ANOVA tests followed by Bonferroni post-tests.

Next we assessed and compared the percentages of these 4 subsets in SAMHD1^low^ and SAMHD1^+^ compartments in both HIV-1 infected and uninfected groups. **Fig 2A** shows the gating strategies used.

Consistently, we found a significant enrichment of Th17 cells in SAMHD1^low^ compartment as compared to Th1Th17, CCR6^+^DP and CCR6^+^DN in both groups (p<0.0001). Percentages in HIV-1^+^ patients were 41% [17.5–62.5] vs 15.3% [3.7–35.5], 7.95% [1.18–22.2] and 5.62% [1.75–17.5]) for Th17 vs Th1Th17, CCR6^+^DP and CCR6^+^DN respectively, P<0.05) **(Fig 2B)**. This trend was similar in uninfected individuals (55% [24.6–76.7] vs 11.8% [3.18–24.6], 8.58% [3.35–19.7] and 3.8% [1.67–8.8]) respectively, P<0.05). Importantly, we observed a significant decrease of %Th17 in SAMHD1^low^ compartment in infected compared to uninfected individuals (41% [17.5–62.5] vs 55% [24.6–76.7], p<0.05), in contrast to CCR6^+^DN cells that increased significantly (7.95% [1.18–22.2] vs 3.8% [1.67–8.8], p<0.05) **Fig 2C).** We have also observed a decreasing trend with no significant differences in the SAMHD1^+^ compartment for Th17 cells between uninfected and infected individuals (25.1% [9.5–47.2] vs 17.9% [6.71–35], p>0.05), while CCR6^+^DN cells increased significantly (24.8% [10.8–35] vs 14.7% [6.38–28.4] p<0.05) **(Fig 2C).** Enrichment and percentages of the 4 CCR6^+^ subsets (DP, DN, Th1Th17, Th17) in SAMHD1^low^ and SAMHD1^+^ compartments in both HIV-1 infected and uninfected groups are delineated in **Fig 2D**.

Altogether, these results point out that Th17 and CCR6^+^DN, two major populations that are susceptible to HIV-1 infection, are enriched in SAMHD1^low^ cells from infected patients.

### Memory CD4^+^CD45RO^+^SAMHD1^low^ cells exhibit the highest level of HIV-1 DNA

Differential expression of CD45RO and SAMHD1 molecules identifies CD45RO^+^SAMHD1^+^, CD45RO^+^SAMHD1^low^ memory and CD45RO^-^SAMHD1^+^ naïve CD4^+^ T-cell subsets [34]. We reasoned that SAMHD1^low^ cells, that are enriched in Th17 and CCR6^+^DN cells would contain high levels of HIV-1 DNA.

**Fig 3:**
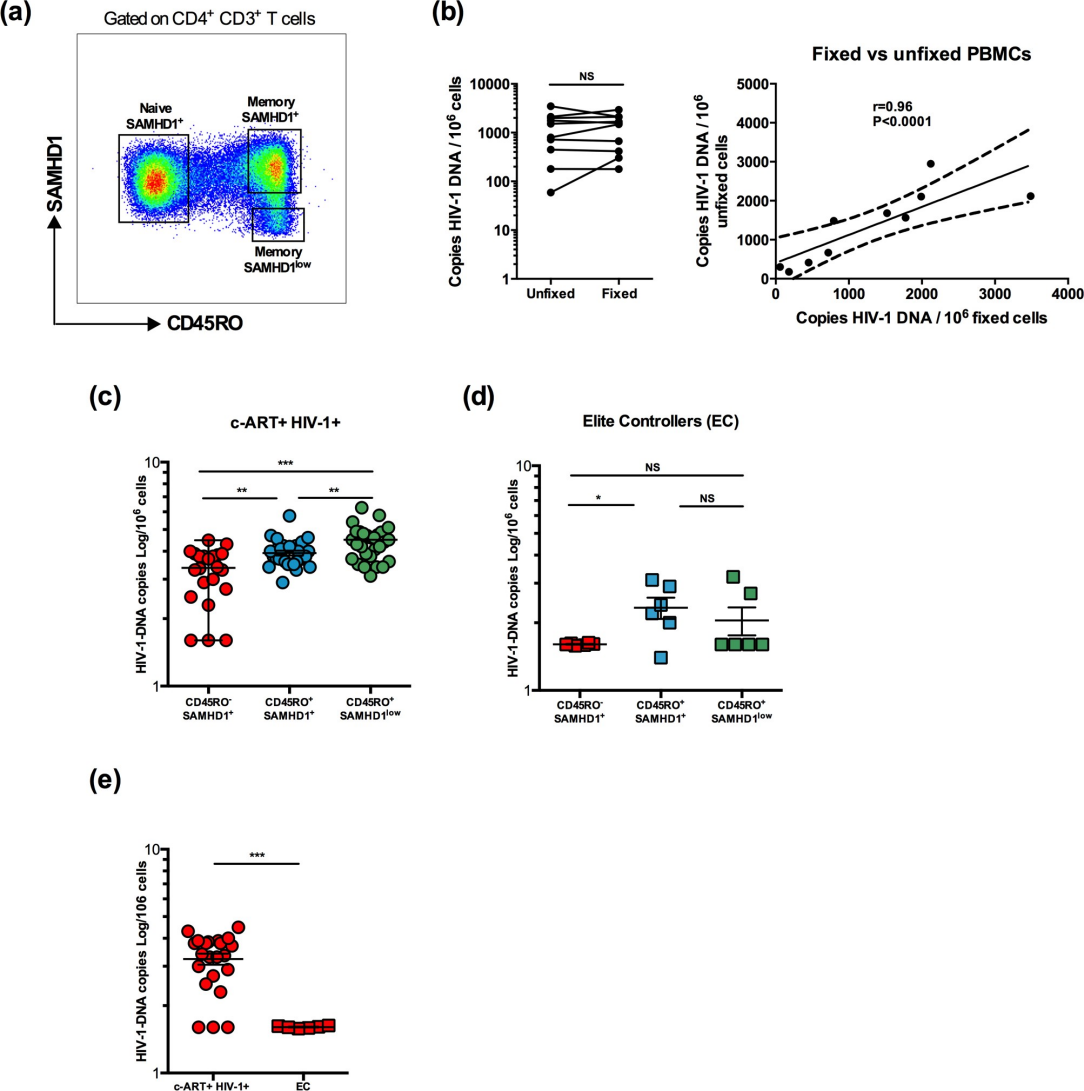
Memory CD4^+^ CD45RO^+^ SAMHD1^low^ cells exhibit the highest level of HIV-1 DNA. Peripheral blood samples were collected from 36 HIV-infected individuals receiving c-ART and 6 Elite Controllers. Fresh PBMCs were stained with anti-CD3, -CD4, -CD45RO and -SAMHD1. The three cell subsets: memory CD4^+^ CD45RO^+^ SAMHD1^low^, CD4^+^ CD45RO^+^ SAMHD1^+^ and naive CD4^+^ CD45RO^-^ SAMHD1^+^ were sorted by FACS. Protocols for DNA extraction and PCR experiments on permeabilized and fixed cells have been optimized using bulk PBMCs from 10 HIV-1 infected individuals receiving c-ART. Levels of total HIV-1 DNA were quantified by Nested real-time PCR in sorted cells *ex vivo*. **(a)** Gating strategy for memory CD4^+^CD45RO^+^SAMHD1^low^, CD4^+^CD45RO^+^SAMHD1^+^ and naive CD4^+^CD45RO^-^SAMHD1^+^. **(b)** Levels of total HIV-1 DNA in unfixed PBMCs from HIV-1 infected individuals receiving c-ART (n = 10) compared head-to-head with fixed PBMCs from the same individuals. **(c)** Levels of total HIV-1 DNA in sorted memory CD4^+^CD45RO^+^SAMHD1^low^, CD4^+^CD45RO^+^SAMHD1^+^ and naive CD4^+^CD45RO^-^SAMHD1^+^ from HIV-1 infected individuals receiving c-ART. **(d)** Levels of total HIV-1 DNA in sorted memory CD4^+^CD45RO^+^SAMHD1^low^, CD4^+^CD45RO^+^SAMHD1^+^ and naive CD4^+^CD45RO^-^SAMHD1^+^ from Elite Controllers (HIV+ EC). **(e)** Levels of total HIV-1 DNA in sorted naive CD4^+^CD45RO^-^SAMHD1^+^ from HIV-1 infected individuals receiving c-ART and Elite Controllers. Data represent median with interquartile range. *p<0.05, **p<0.01 and ***p<0.001, t-test and 2-way ANOVA tests followed by Bonferroni post-tests.

In order to quantify HIV-1 DNA, CD45RO^+^ SAMHD1^low^ and SAMHD1^+^ memory and CD45RO^-^SAMHD1^+^ naïve cells were FACS-sorted based on their SAMHD1 expression (low or high) **(Fig 3A)**. Given that cells required fixation and permeabilization for staining, we developed suitable protocols for nucleic-acids extraction and quantitative PCR experiments for HIV-1 quantification as detailed in the methods part.

We first trialed our new protocol using unfixed and fixed/permeabilized peripheral blood mononuclear cells (PBMCs) to quantify and compare levels of total HIV-1 DNA in 10 HIV-1 infected individuals receiving c-ART, with a limit of detection of 3 copies/PCR. We expressed each value as number of HIV-1 DNA copy per million of cells. The results indicated that fixed and unfixed PBMCs harboured HIV-1 DNA at equivalent levels (**Fig 3B** left panel; median: 1524.07 [179–2947.2] vs 1159.48 [59.31–3487] copies/10^6^ cells, respectively, p = 0.6520). Importantly, HIV-1 DNA copy numbers in fixed and unfixed PBMCs were significantly correlated, thus showing the reliability of our protocol (r = 0.96, p<0.0001, **Fig 3B** right panel).

We next determined whether SAMHD1^low^ cells harboured highest HIV-1 DNA levels in comparison to SAMHD1^+^ from infected individuals under c-ART (n = 36). We found that memory SAMHD1^low^ cells from c-ART HIV-1 infected individuals harboured high levels of HIV-1 DNA compared to memory SAMHD1^+^ cells (4.5 [3.1–6.2] vs 3.8 [2.9–5.7] log/10^6^ cells, respectively, p = 0.009), while naïve SAMHD1^+^ showed significantly lower levels (3.1 [1.6–4.4], p<0.0001 and p = 0.006 vs memory SAMHD1^low^ and SAMHD1^+^ respectively) **(Fig 3D)**. In HIV-1 infected elite controllers group (EC, n = 6), both memory SAMHD1^low^ and SAMHD1^+^ cells harboured low levels of HIV-1 DNA as anticipated (1.6 and 2.3 log/10^6^ cells, respectively, p>0.05) **(Fig 3E)**. Naïve SAMHD1^+^ cells from EC also showed lower DNA compared to naïve SAMHD1^+^ cells from c-ART HIV-1 infected individuals (1.6 and 3.1 log/10^6^ cells, respectively, p = 0.001) **(Fig 3F)**. Of note, there was no association between levels of HIV-1 DNA and patients’ clinical characteristics (gender, age, duration under c-ART, nadir CD4). Altogether, these results identify memory SAMHD1^low^ CD4^+^ T cells as a novel cell subset harbouring high levels of HIV-1 DNA among memory cells, thus contributing to HIV-1 reservoir.

### HIV-1 viral populations are differentially compartmentalized in memory SAMHD1^low^ and SAMHD1^+^ cells

It is well known that HIV-1 infects and rapidly disseminates in CD4^+^ T cells representing thus a reservoir that will seed other cell compartments and tissues as soon as c-ART is stopped [36]. We reasoned that HIV-1 sequences identification and comparison across memory SAMHD1^low^ and SAMHD1^+^ as well as naïve SAMHD1^+^ subsets, will provide important information on viral exchange and spread between CD4^+^ subsets.

To reach this goal, we performed ultra-deep-sequencing (UDS, 454/Roche) of partial HIV-1 *env* (C2/V3) DNA, obtained from sorted CD45RO^+^SAMHD1^+^ CD45RO^+^SAMHD1^low^ and naïve CD45RO^-^SAMHD1^+^ CD4^+^ T-cells of 12 HIV-1 infected individuals receiving c-ART. We chose env as this gene is essential for HIV-1 replication-competence. Also, env is the most diverse gene within HIV-1 genome, therefore focusing on this gene can bring to light any small difference in viral populations amongst the different studied cell subsets. Of note, we did not obtain enough DNA on sorted CD4^+^CD45RO^+^SAMHD1^low^ cells from patient #9.

**Fig 4:**
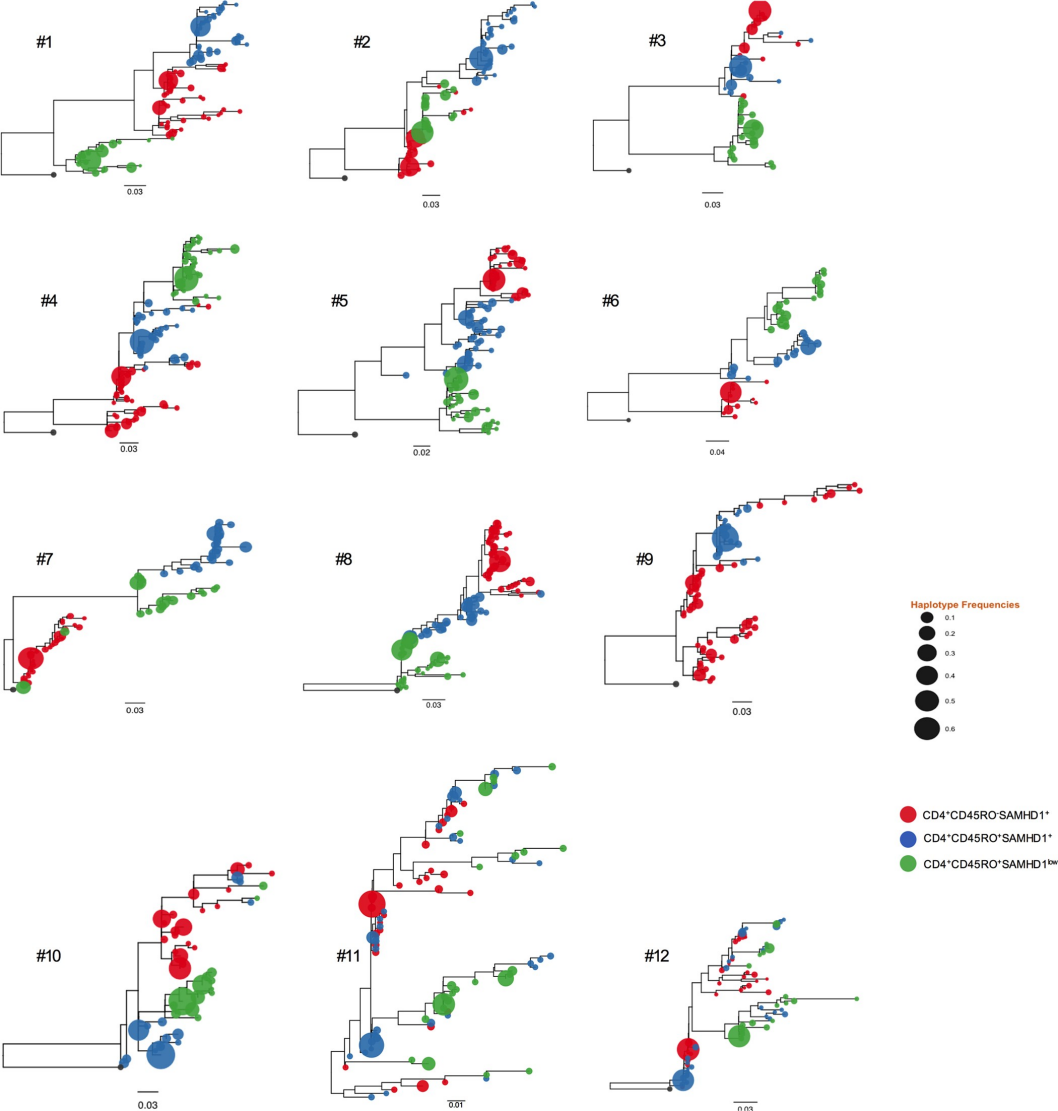
Distinct/well-segregated HIV-1 DNA populations in both memory CD4^+^ CD45RO^+^ SAMHD1^low^, CD4^+^ CD45RO^+^ SAMHD1^+^ and naive CD4^+^ CD45RO^-^ SAMHD1^+^ subsets. Ultra-deep-sequencing (UDS, 454/Roche) of partial *env* (C2/V3) HIV-1 DNA (n = 12 HIV-1 infected individuals receiving c-ART) was performed. Neighbor-joining phylogenetic trees representing compartmentalized and equilibrated viral populations in 10 patients out of 12 studied patients. Genetic distance is indicated with a scale bar (number of nucleotide substitutions per site). Shown are results for the 12 individuals. HIV haplotypes were extracted from reads covering partial *env*; e*nv* sequences from naive CD4^+^CD45RO^-^SAMHD1^+^, memory CD4^+^CD45RO^+^SAMHD1^+^ and CD4^+^CD45RO^+^SAMHD1^low^ are depicted in red, blue, and green, respectively. Tip size is proportional to variant frequency. Scale bars are in substitutions/site.

Phylogenetic analyses revealed well-segregated HIV-1 DNA populations between memory SAMHD1^low^ and SAMHD1^+^, and naïve SAMHD1^+^ CD4^+^ T-cells subsets (p<0.001) in all but 2 individuals (#11 & #12 individuals). Next, we applied non-parametric test for population structure to all participants for each cellular subset. When assessing viral population structure across all subsets, FST and Slatkin Maddison approaches confirmed significant compartmentalization between memory SAMHD1^low^ and SAMHD1^+^ cells in 10 samples (**Fig 4**) but 2 (#11 & #12 individuals). The remaining 2 individuals did not exhibit significant signals for compartmentalization represented by non-homogeneous distribution of the three populations **(Fig 4**).

To quantify the diversity of HIV-1 *env* in HIV population originating from different subsets, we computed the average pairwise distance (APD) between reads in all samples from the 12 studied individuals. We also determined the genetic divergence (i.e. APD between reads) from different cellular subsets within host.

Every individual showed distinct degrees of HIV-1 DNA diversity within subsets and divergence between subsets **(S1 Fig)**. The APD between subsets (i.e. divergence) confirmed the well-segregated HIV-1 DNA populations in subsets, suggesting a very limited viral exchange. We then used Slatkin Maddison counts to provide a quantitative estimate of viral gene flow between each cell subset **(S2 Fig)**. We confirmed the minimal viral exchange represented by low number of migration events (ME) originating from memory SAMHD1^low^ toward naïve SAMHD1^+^ and memory SAMHD1^+^ cell subsets (4.44ME [1–8] and 2.25ME [1–4], respectively), from memory SAMHD1^+^ cells toward naïve SAMHD1^+^ and memory SAMHD1^low^ cell subsets (4ME [1–22] and 4.62ME [1–12], respectively), and from naïve SAMHD1^+^ toward memory SAMHD1^+^ and SAMHD1^low^ cell subsets (8ME [2–16] and 5.77ME [1–18], respectively).

Moreover, we conducted phylogenetic analyses and a large variety of tree-based (i.e. Slatkin Maddison (SM), Simmonds association index (AI), Fitch parsimony score (PS), and monophyletic clade size (MC)) and distance-based methods (Wright’s measure of population subdivision, i.e. FST test) to investigate the population structure and dynamics of HIV-1 reservoir and elucidate cellular processes that maintain it. Based on the SM, F_ST_, AI, and PS statistics, we rejected the null hypothesis of no association between the location trait (cellular subset) and the phylogeny (*P*<0.001) (**S1 Table**). For the MC statistic, we also rejected the null hypothesis of no association between sampled location and the structure of the phylogeny (*P*≤0.01) for all but 2 participants (#11 and #12).

### SAMHD1^low^ cells contain p24-producing cells

**Fig 5:**
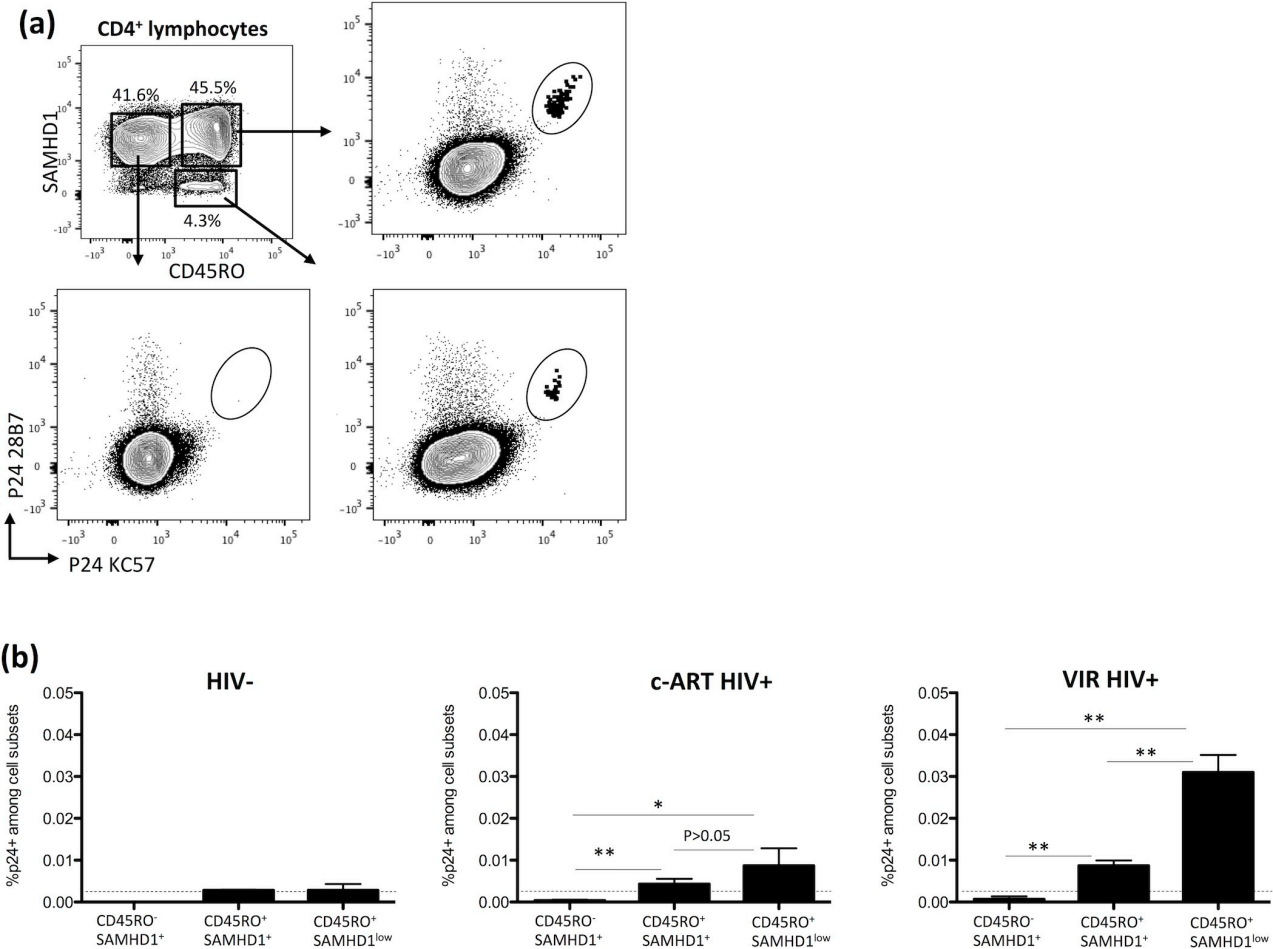
Detection of p24-producing cells in memory CD4^+^ CD45RO^+^ SAMHD1^low^, CD4^+^ CD45RO^+^ SAMHD1^+^ and naive CD4^+^ CD45RO^-^ SAMHD1^+^ subsets. Samples from 3 viremic (VIR) and 7 c-ART individuals were used. 3 HIV-negative control were included. **(a)** Representative dot plots showing the p24 KC57-PE / p24 28B7-APC co-staining in purified CD4^+^ T cells stimulated with anti-CD3/CD28 antibodies for 48h. P24^+^ cells in memory CD4^+^ CD45RO^+^ SAMHD1^low^, CD4^+^ CD45RO^+^ SAMHD1^+^ and naive CD4^+^ CD45RO^-^ SAMHD1^+^ subsets are shown **(b)** Frequencies of p24^+^ cells in each subset from c-ART and VIR HIV^+^ individuals are represented by histograms. Data represent median with interquartile range. Unpaired t test was applied for comparison between two subsets; *p<0.05, **p<0.01. Dotted-line points to the non-specific staining observed in HIV- individuals.

We next asked whether HIV-1 contained in memory SAMHD1^low^ and SAMHD1^+^ CD4^+^ T-cells has the capacity to replicate contributing thus to the stability of HIV-1 reservoirs. We used a flow cytometry based assay recently developed by Chomont and colleagues named HIV-Flow [37], to simultaneously quantify and characterize HIV-1 reservoir. By combining two antibodies targeting the HIV-1 capsid (KC57 and 28B7), we were able to detect the presence of p24-producing cells in stimulated memory SAMHD1^+^ and SAMHD1^low^ cell subsets from both c-ART and viremic HIV^+^ individuals. This patients cohort is different from the one described above. In **Fig 5A** we represented an example of the gating strategy used to identify and quantify p24^+^ cells in each subset. Of note, we did not observe any significant difference in SAMHD1 and CD45RO expressions after 48hrs *in vitro* stimulation of purified CD4^+^ T cells. We compiled data from 7 c-ART HIV^+^ and 3 viremic (VIR) HIV^+^ individuals including 3 HIV- as controls (**Fig 5B**). We found a higher frequency of p24^+^ cells in SAMHD1^low^ compared to SAMHD1^+^ memory cells in c-ART (mean: 0.01% vs 0.005%) and VIR HIV^+^ (mean: 0.03% vs 0.008%) individuals. This difference was significant for VIR HIV^+^ (p<0.01). We detected very low levels of non-specific staining in HIV- individuals as compared to HIV^+^. This background is represented by a dotted line (**Fig 5**). When we calculated the ratio p24^+^ in SAMHD1^low^: p24^+^ in SAMHD1^+^ cells we found it higher in VIR HIV^+^ as compared to c-ART HIV^+^ patients (3.75 vs 2). These data demonstrate that SAMHD1^low^ cells from VIR HIV^+^ patients contain a greater number of competent virus. Altogether, these results demonstrate that SAMHD1^low^ cells represent a factual reservoir for HIV-1.

### HIV-1 DNA levels positively correlated with Ki67 expression in memory SAMHD1^low^ cells

In our previous study we reported that peripheral SAMHD1^low^ CD4^+^ T cells were enriched in cycling cells [34]. Here, we evaluated Ki67 expression in patients and assessed the relationship between Ki67 and HIV-1 DNA in memory SAMHD1^low^ and SAMHD1^+^ CD4^+^ cells.

**Fig 6:**
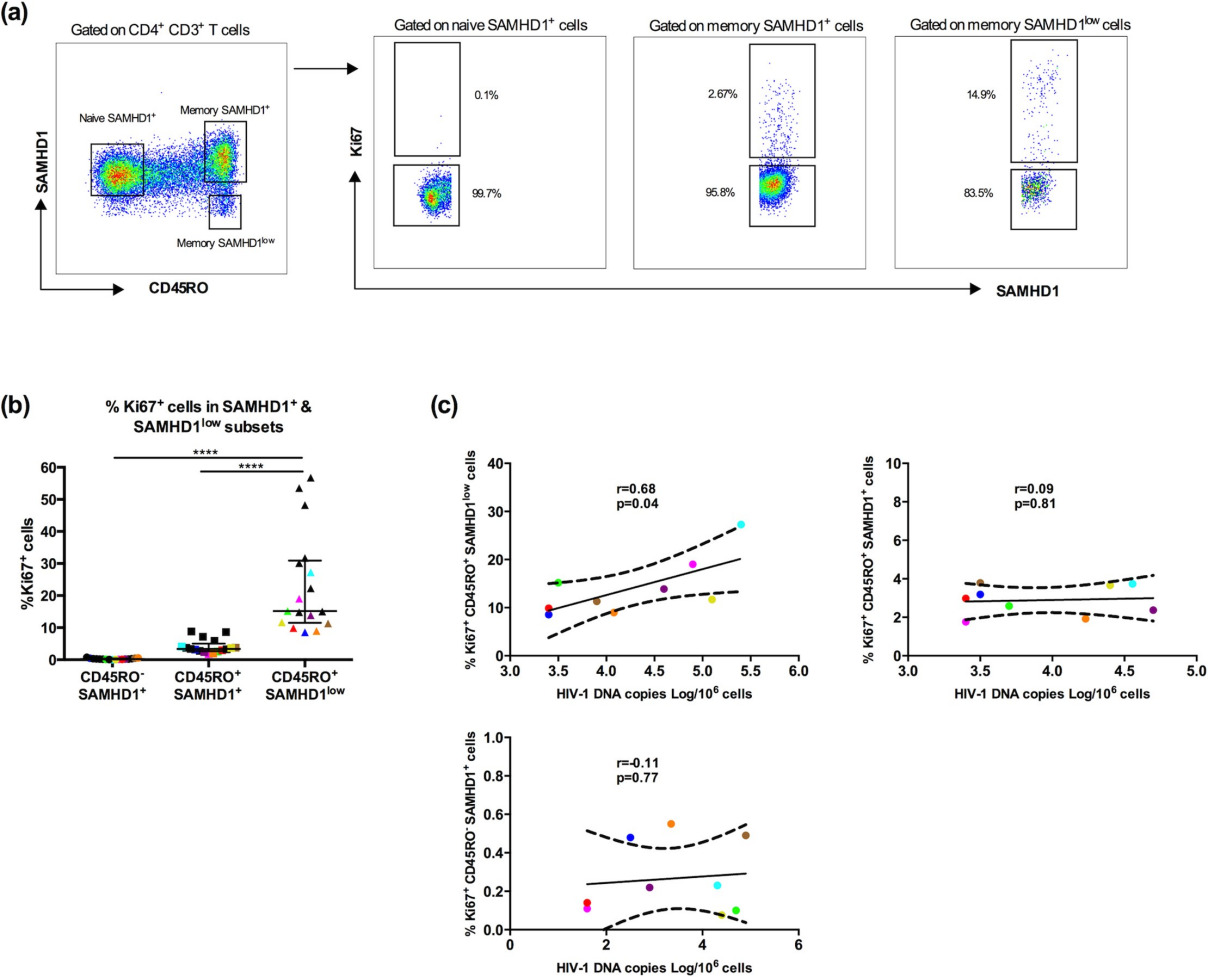
Total HIV-1 DNA levels positively correlate with Ki67 expression in memory SAMHD1^low^ cells. Frozen PBMCs from HIV-1 infected individuals receiving c-ART (cART^+^ HIV-1^+^, n = 17) were stained with a cocktail of fluorochrome-conjugated -CD3, -CD4, -CD45RO, and -SAMHD1 monoclonal antibodies. A viability staining was used to exclude dead cells. **(a)** Gating strategy for Ki67^-^ cells among naïve SAMHD1^+^ (CD45RO^-^ SAMHD1^+^), memory SAMHD1^+^ (CD45RO^+^SAMHD1^+^) and memory SAMHD1^low^ (CD45RO^+^SAMHD1^low^) compartments. **(b)** Percentages of Ki67^+^ cells amongst SAMHD1^+^ and SAMHD1^low^ CD4^+^ T cells. Colored symbols represent the nine patients shown in (c). **(c)** Correlations between HIV-1 DNA levels and Ki67 expression of naïve SAMHD1^+^ (CD45RO^-^ SAMHD1^+^), memory SAMHD1^+^ (CD45RO^+^SAMHD1^+^) and memory SAMHD1^low^ (CD45RO^+^SAMHD1^low^) cells (n = 9 patients). Data represent median with interquartile range and ****p<0.001, t-test and 2-way ANOVA tests followed by Bonferroni post-tests and Spearman correlation.

We first investigated the expression of Ki67 in c-ART HIV-1 infected individuals. As expected and as previously shown [34], we confirmed that %Ki67^+^ cells were significantly higher in memory SAMHD1^low^ cells compared to memory SAMHD1^+^ and naïve SAMHD1^+^ cells (15.2% [8.56–56.8] vs 3.37% [1.76–8.81] and 0.23% [0–0.79] respectively, p<0.0001) (**Fig 6A and 6B**). We next assessed the relationship between Ki67 expression and levels of HIV-1 DNA in SAMHD1^low^ and SAMHD1^+^ memory and SAMHD1^+^ naïve cells in 9 patients. While there was no correlation between HIV-1 DNA and Ki67 expression in memory SAMHD1^+^ and naïve SAMHD1^+^ CD4^+^ T-cells (**Fig 6C** top right and bottom panels, r = +0.09 and r = +0.11 respectively, p>0.05), we observed a statistically significant positive association between levels of HIV-1 DNA and Ki67^+^ in memory SAMHD1^low^ cells (r = +0.68, p<0.05) **(Fig 6C**, left top panel).

Altogether, these results suggest that Ki67^+^SAMHD1^low^ cells represent a reservoir for HIV-1.

## Discussion

Expanded CD4^+^ T cells with integrated HIV-1 can be a hurdle for achieving a cure as these cells represent a long-lived and persistent reservoir for the virus [38].

The goal of this study was to investigate whether the highly proliferative memory SAMHD1^low^ CD4^+^ T cells, previously identified by our group as being permissive to HIV-1 and significantly depleted during infection [34], contribute to HIV-1 persistence by potentially replenishing the reservoir. To this end we performed HIV-1 DNA and p24-producing cells quantification together with HIV-1 sequencing in sorted CD4^+^ memory CD45RO^+^SAMHD1^low^, CD45RO^+^SAMHD1^+^ cells and naïve CD45RO^-^SAMHD1^+^ cells from HIV-1 infected individuals and we characterized their phenotype. Our results demonstrate that (i) Th17 and CCR6^+^DN-expressing transcriptional signature of early Th17 [18,35] two major populations that are susceptible to HIV-1 infection, are present in SAMHD1^low^ cells, and while the former decreased significantly in c-ART HIV-1 infected compared to uninfected individuals, the latter significantly increased; (ii) memory SAMHD1^low^ cells from c-ART patients carry high levels of HIV-1 DNA compared to SAMHD1^+^ cells, and these levels positively and significantly correlated with Ki67 expression; (iii) SAMHD1^low^ cells contain p24-producing cells; (iv) phylogenetic analyses revealed well-segregated HIV-1 DNA populations with significant compartmentalization between SAMHD1^low^ and SAMHD1^+^ cells and limited viral exchange. Altogether these data demonstrate that memory SAMHD1^low^ cells are a reservoir thus contributing to HIV-1 persistence.

It is well known that persistence of long-lived and latently infected resting memory cells in c-ART patients, represents the major barrier to cure [1,39–46]. It has been suggested that the pool of latently infected cells is not static and HIV-1 reservoir can persist through cell proliferation, whether it is homeostatic- or TCR- induced [2,47,48], but also through provirus integration into cellular genes associated with cell survival and/or proliferation [48,49]. Thus low-viral replication leads to de novo cellular infection and constant replenishment of HIV-1 reservoir.

Based on our recent data where we demonstrated that SAMHD1 levels decreased with cell proliferation, which was associated with higher susceptibility to HIV-1 infection *in vitro* [34], we reasoned that memory SAMHD1^low^ cells from c-ART infected patients harbour high levels of HIV-1 DNA that correlate with their proliferative capacity. Indeed we found that patients’ memory SAMHD1^low^ cells harboured significant high levels of HIV-1 DNA as compared to memory SAMHD1^+^ cells and expressed increased Ki67 levels, which confirms previous data obtained with a different cohort of c-ART infected patients [34]. Most importantly, we observed a statistically significant positive association between levels of HIV-1 DNA and Ki67 expression in memory SAMHD1^low^ cells (r = +0.68, p<0.05), while there was no correlation between HIV-1 DNA and Ki67 expression in memory SAMHD1^+^, which suggest that proliferative SAMHD1^low^ CD4^+^ contribute to HIV-1 persistence. Most importantly, we showed that SAMHD1^low^ cells contain p24-producing cells, which demonstrates that these cells represent a reservoir for the virus. Our data are in accordance with a recent report where it has been elegantly shown that HIV-1 targets host DNA in a cell-cycle dependent manner [50].

We next used ultra-deep sequencing to genetically characterize HIV-1 DNA populations from sorted memory SAMHD1^low^, SAMHD1^+^ and naive SAMHD1^+^. We conducted phylogenetics analyses and a large variety of tree-based and distance-based methods to investigate the population structure and dynamics of HIV-1 reservoir and elucidate cellular processes that maintain it. Altogether, our data revealed well–segregated HIV-1 DNA populations and cell-specific compartmentalization of HIV-1 DNA populations across the three cell subsets, in 10 out of 12 individuals. The compartmentalization between memory and naïve CD4^+^ T cells is consistent with previous studies where distinct HIV-1 DNA populations were observed in various cell subsets including total memory, activated and naïve CD4^+^, blood monocytes and NK cells [8,9,51]. Importantly, we detected sustained compartmentalization between memory SAMHD1^+^ and memory SAMHD1^low^ cells revealing for the first time compartmentalization within the same cell memory pool, i.e cells with differential SAMHD1 expression. Moreover, we observed limited viral exchange (i.e. Slatkin Maddison migration events, S2 Fig) originating from naïve SAMHD1^+^, predominantly directed toward memory SAMHD1^+^ cell subset (p >0.05).

The limited sample size and sequenced HIV-1 coding region (partial HIV-1 *env*) prevent us to rule out that viral exchange may occur between cellular subsets, with other treatment regimens or in other populations. Another limitation of this report is the use of the 454 sequencing platform, which is more prone to homopolymer-associated, per-base errors [52] and artifact recombination [53]. To overcome this limitation, we applied rigorous quality control procedures for deep sequencing, as previously described in our group and others [54–56]. Our validated bioinformatics pipeline includes strict quality filtering steps [57–59] and only quality-controlled reads were included in the analysis [60]. Future studies using more recent sequencing technologies that are less prone to single-base errors, the use of Primer ID [61] or single genome amplification/Sanger sequencing [62] in different settings could also be enlightening.

Nevertheless, to our knowledge this is the first study revealing such compartmentalization in viral sequences between cells from the same memory pool.

In our previous study, we reported that peripheral blood memory SAMHD1^low^ CD4^+^ T cells were enriched in CXCR3^-^CCR6^+^CCR4^+^ Th17 cells and were depleted in HIV-1 infected individuals [34]. Th17 cells are presumed to be the most susceptible cells to HIV-1 infection and are preferentially depleted in infected individuals [16,17, 63–67]. Recently, Wacleche et al. revealed the existence of two novel CCR6^+^ subsets: CCR6^+^DP cells that co-express CXCR3 and CCR4 [18,35], and CCR6^+^DN cells that lack both molecules, express a unique transcriptional signature of early Th17 and Tfh, are able to proliferate in response to IL-2 and contain replication competent HIV-1. Importantly, this group showed that in contrast to the classical CCR6^+^ subsets (Th17 and Th17Th1) that were depleted in infected individuals, CCR6^+^DN cells were not [18,35]. Remarkably, we found that CCR6^+^DN cells were preserved in SAMHD1^low^ cells from infected compared to uninfected individuals (8% vs 3.8%), while Th17 SAMHD1^low^ cells were significantly decreased (41% vs 55%, p<0.05). Altogether these data demonstrate that SAMHD1^low^ cells are enriched in Th17 and CCR6^+^DN cells, two major targets and contributors to HIV-1 persistence in peripheral blood.

Moreover, we observed that in contrast to SAMHD1^low^ cells, memory SAMHD1^+^ cells contain less Th17 in both infected and uninfected individuals (41% vs 15.4% and 55% vs 25%, respectively) and their depletion during infection was non-significant (Fig 2C). However, CCR6^+^DN were more abundant in SAMHD1^+^ than in SAMHD1^low^ (25.6% vs 8% and 14.7% vs 3.8%, respectively), which let us hypothesize that it might be partly due to high SAMHD1 expression, consistent with its role as a restriction factor that inhibits viral life cycle and cell infection and depletion [26–29].

Previous work suggested a role of homeostatic proliferation as a cause of persistence [2,68]. Cytokines such as interleukin 7 (IL-7) and/or interleukin 2 (IL-2), are known to enhance the survival and proliferation of memory CD4^+^ T cells [2,69,70] and IL-7 has also been shown to induce proliferation of latently infected cells without viral reactivation or genetic diversification [2,71]. The fact that SAMHD1^low^ cells showed high proliferative capacity *in vivo*, which was positively correlated with HIV-1 DNA, suggested to us that they could be sensitive to IL-7 and/or IL-2 induced-homeostatic proliferation. To this end, we assessed IL-7 receptor alpha-chain (CD127) and IL-2 receptor alpha-chain (CD25) expressions in *ex vivo* memory SAMHD1^low^ and SAMHD1^+^ cells from infected and uninfected individuals. Our data revealed higher CD25 and lower CD127 expressions in SAMHD1^low^ cells compared to SAMHD1^+^ (p<0.05), suggesting a role of these cytokines in homeostatic proliferation. Indeed, these observations need to be confirmed.

We have previously shown that the down-regulation of SAMHD1 expression during T-cell activation and differentiation renders CD4^+^ T cells susceptible to HIV-1 infection [34]. Here we show that SAMHD1^low^ memory cells display the highest levels of HIV-1 DNA and contain p24-producing cells, which is in relation to their higher susceptibility to depletion in infected patients. Of note, SAMHD1^low^ cells include Th17 and Tfh cells, the main targets of HIV-1 [21,25]. Moreover, it is interesting to consider that despite similar CD4 and CCR5 expressions in SAMHD1^low^ and SAMHD1^+^ cells (**S3 Fig**, **S4 Fig**), we found more HIV-1 DNA and p24^+^ cells in SAMHD1^low^ cells, which might be the consequence of post-entry mechanisms.

We can reason that HIV-1 persistence in proliferating SAMHD1^low^ memory T cells is facilitated by the lack of SAMHD1. The lack of this restriction factor relieves the pressure on the virus allowing thus its integration and possible compartmentalization following proliferation. These results uncover a new mechanism that may account for the high susceptibility toward HIV-1 infection of rapidly proliferating effector/ memory CD4^+^ T cells.

The rare viral exchange observed between different CD4^+^ T cell subsets from the blood is rather surprising, as memory CD4^+^ T cells arise from naive cells. Indeed, SAMHD1^low^ memory cells may most likely arise from activation of either naive or memory SAMHD1^+^ CD4^+^ T cells. However, the compartmentalization of HIV-1 sequences suggests that SAMHD1^low^ may be infected following their activation rather than derived from infected naive or memory SAMHD1^+^ T cells. Importantly, as our cohort of HIV-1 patients are under c-ART and have undetectable viremia, our data provide another evidence of residual HIV-1 replication in patients under successful therapy as previously shown [72]. Future work would be required to confirm whether this residual replication occurs in lymphoid tissues or in the gut, which contain the majority of HIV-1 infected CD4^+^ T cells. Longitudinal studies should also provide evidence on the fate of SAMHD1^low^ memory cells and the stability of SAMHD1^low^ reservoir and further characterize the dynamics of viral exchange between CD4^+^ T cell subsets. As SAMHD1^low^ memory cells are also cycling, they might participate to the residual HIV-1 replication. The advance in high dimensional technics such as single cell RNA sequencing would also bring information on whether SAMHD1^low^ memory CD4^+^ T cells contribute to this residual viral replication.

This study provides valuable information for exploring the dynamics of HIV-1 in memory CD4^+^ T cell compartment and reveal that proliferation, which characterizes CD4^+^ SAMHD1^low^ memory cells (pool of Th17, Tfh and Treg [34]), could be targeted for HIV cure, thus opening new avenues for HIV-1 research.

## Materials and methods

### Ethics statement

Ethics committee (CCPPRB Créteil Henri Mondor) approved this study and written informed consent from all patients, in accordance with the Declaration of Helsinki, were obtained prior to study initiation.

### Biological samples

Peripheral blood was collected from healthy controls (n = 13) obtained from Etablissement Français du Sang (Créteil, France) and from c-ART HIV-1 infected individuals (n = 49), were recruited at the Department of Infectious Diseases in Henri Mondor Hospital (Créteil, France). Patients from this cohort were used for phenotypic characterization and/or HIV-1 DNA quantification and sequencing. HIV-1 infected elite controllers (EC, n = 6) were included in some experiments. Tables 1 and 2 summarize patients’ characteristics; for HIV-1 treated patients, [median (range) CD4^+^ T-cell count: 572 (129–1393) cells/ml; HIV-1 RNA: <20 (0–7404) copies/ml]; for EC (CD4^+^ count: 900 cells/μl).

**Table 1:** c-ART and VIR HIV^+^ patients.

	Characteristics of the study population								
N Pt	Gender	Age	Duration of treatment (years)	Time from HIV-1+	CD4 count/ml	CD8 count/ml	CD3 count/ml	VL (copies/ml)	Nadir CD4
**1**	M	65	4	**15**	471	414	892	<20	319
**2**	M	75	18	**26**	657	509	1165	<20	158
**3**	M	74	16	**22**	590	581	1238	<20	254
**4**	F	45	15	**20**	893	1140	2018	<20	212
**5**	F	37	1	**5**	431	548	991	<20	210
**6**	F	52	16	**21**	1393	1009	2410	<20	237
**7**	F	39	10	**14**	347	403	813	<20	167
**8**	F	40	9	**16**	619	417	1036	<20	137
**9**	M	57	19	**27**	621	567	1212	<20	155
**10**	F	51	20	**24**	549	413	978	<20	274
**11**	M	74	18	**22**	458	511	972	<20	218
**12**	F	58	13	**17**	1229	1203	/	<20	389
**13**	M	69	11	**18**	860	1257	2089	<20	162
**14**	M	67	12	**16**	741	533	1306	<20	156
**15**	M	61	34	**21**	311	242	579	<20	179
**16**	M	74	17	**22**	500	586	1138	<20	254
**17**	F	45	16	**20**	643	781	1425	<20	212
**18**	M	69	20	**32**	587	575	1158	22	100
**19**	F	51	8	**13**	606	315	997	<20	108
**20**	M	42	3	**11**	385	905	1329	<20	220
**21**	F	40	7	**16**	604	486	1097	<20	137
**22**	M	51	3	**17**	436	657	1110	<20	310
**23**	M	49	16	**23**	522	823	1371	<20	178
**24**	M	62	13	**20**	596	467	1057	<20	174
**25**	F	60	21	**31**	774	583	1378	<20	169
**26**	M	66	10	**16**	601	1125	1712	<20	45
**27**	F	72	14	**24**	1080	469	1554	<20	528
**28**	M	69	18	**32**	499	518	1022	<20	100
**29**	M	60	16	**29**	740	/	/	<20	250
**30**	F	65	6	**12**	558	523	1101	<20	30
**31**	F	34	1	**5**	550	267	862	<20	367
**32**	M	37	14	**19**	269	1024	1340	<20	176
**33**	F	56	11	**15**	951	1124	2063	<20	185
**34**	M	57	18	**30**	/	/	/	/	175
**35**	F	72	18	**26**	1357	627	2039	<20	545
**36**	M	41	1	**5**	423	321	772	<20	350
**37**	F	57	20	**22**	398	522	979	<20	135
**38**	M	57	7	**8**	129	789	990	<20	4
**39**	M	41	12	**13**	490	668	1213	<20	114
**40**	M	52	9	**/**	401	ND	ND	<20	399
**41**	M	49	6	**9**	139	689	845	<20	107
**42**	M	49	23	**25**	633	ND	ND	<20	343
**43**	M	49	8	**17**	436	657	1110	<20	310
**44**	M	45	21	**23**	522	823	1371	<20	178
**45**	M	38	8	**11**	385	905	1329	<20	220
**46**	F	36	14	**18**	666	1130	1839	86	193
**47**	M	49	23	**26**	714	417	1139	<20	261
**48**	F	61	14	**19**	879	697	1582	<20	209
**49**	M	53	21	**30**	418	835	1275	<20	175
**50**	F	58	17	**17**	793	901	1731	<20	222
**51**	F	40	13	**13**	747	763	1142	<20	114
**52**	M	36	8	**10**	460	363	850	<20	233
**53**	M	55	24	**26**	756	436	1237	<20	261
**54**	M	55	22	**22**	1020	624	1654	<20	191
**55**	M	46	5	**6**	489	997	1620	<20	297
**56**	M	53	20	**20**	664	393	1072	<20	210
**57**	M	40	none	**1month**	325	806	1142	27000	325
**58**	F	31	none	**/**	468	606	1096	101	510
**59**	F	32	none	**/**	199	263	484	5647	199

**Table 2:** EC patients.

	Characteristics of the study population								
N Pt	Gender	Age	Time from HIV-1+	CD4 Count/ml	CD8 Count/ml	CD3 Count/ml	VL (copies/ml)	Treatment	Nadir CD4
**1**	M	48	33	783	1220	2172	244	naif	332
**2**	F	52	33	1129	394	1548	0	naif	1106
**3**	F	45	29	943	675	1638	308	naif	441
**4**	F	33	17	737	337	1123	0	naif	687
**5**	F	55	34	1483	875	2520	188	naif	1110
**6**	M	26	6	857	994	1941	0	naif	628

### *ex-vivo* T-cell phenotyping

Fresh PBMCs were isolated by density gradient centrifugation. FACS staining was carried out in PBS 1X supplemented with 2% FBS (foetal bovine serum) for 30 min at 4°C using the following fluorochrome-conjugated antibodies: APC-Vio770 anti-CD4, PerCP-Cy5.5 anti-CD3 (Milteniy Biotech, Paris, France), PE-CF594 anti-CD45RO, Alexa-Fluor 647 anti-CCR4, PE anti-CXCR3, PE-Cy7 anti-CCR6 (Becton Dickinson Biosciences, Pont de Claix, France). Intracellular staining was carried out using the FoxP3 permeabilization solution kit according to the manufacturer’s instructions (eBioscience, Paris, France) together with Alexa-Fluor 488 anti-SAMHD1 (from O. Schwartz’s lab; Institut Pasteur, Paris) and PE anti-Ki67 (Becton Dickinson). Dead cells were excluded using the Live/Death Vivid detection kit labeled with an aqua dye (Invitrogen). Fluorescence intensities were measured with an LSR II flow cytometer (Becton Dickinson) and analyzed using FlowJo version 10.0.7 (Tree Star Inc., Ashland, Oregon, USA).

### HIV-flow procedure

Fresh and frozen samples from 7 c-ART, 3 viremic (VIR) HIV^+^ and 3 healthy HIV- individuals were included. CD4^+^ T cells were isolated by negative selection using magnetic beads (Miltenyi Biotech). Purity was >95%. 3-10x10^6^ CD4^+^ T cells, depending on patients’ CD4 count, were resuspended at 2x10^6^ cells/mL in RPMI + 10% Fetal Bovine Serum. Cells were stimulated with 0.5ug/ml anti-CD3/CD28 antibodies (Miltenyi Biotech) for 48hrs at 37°C. Samples were pre-incubated for 1h with 5μg/mL Brefeldin A (BFA, Sigma, B2651) before stimulation. BFA was maintained in the culture until the end of the stimulation. After stimulation, cells were collected, resuspended in PBS and stained with the Aqua Live/Dead staining kit for 30min at 4°C. Cells were then stained with antibodies against cell surface molecules: APC-Vio770 anti-CD4 (Miltenyi Biotech), Pacific Blue anti-CD3 and PE-CF594 anti-CD45RO (BD Bioscience) in PBS + 4% human serum (Institut de Biotechnologies Jacques Boy, France) for 30min at 4°C. Cells were fixed and permeabilized using the “FoxP3 permeabilization solution kit” according to the manufacturer’s instructions (Affymetrix, eBioscience), and then stained with PE anti-p24 KC57 (Beckman Coulter, Villepinte France), APC anti-p24 28B7 (1/10 dilution) (Interchim, Montluçon France) and Alexa-Fluor 488 anti-SAMHD1 for an additional 45min at RT. The frequency of p24 double positive cells (KC57^+^, 28B7^+^) was determined by flow cytometry (BD LSRII) in gated viable CD4 T cells.

### Cell sorting

Fresh peripheral blood mononuclear cells (PBMCs) were isolated by density gradient centrifugation. CD4^+^ T-cells were separated from PBMCs using the “CD4 T cell Isolation kit II” (Miltenyi Biotech) according to the manufacturer’s instructions.

Staining was performed on fresh samples, directly following PBMCs collection and CD4^+^ T-cells isolation. Cells were stained with a cocktail of fluorochrome-conjugated antibodies for 30 min at 4°C: APC-Vio770 anti-CD4 (Miltenyi Biotech), Pacific Blue anti-CD3 and PE-CF594 anti-CD45RO (BD Bioscience). Following surface staining, cells were washed once in PBS 1X/ 2% FBS and fixed/permeabilized using the “FoxP3 permeabilization solution kit” according to the manufacturer’s instructions (Affymetrix, eBioscience). Intracellular staining was performed using Alexa-Fluor 488 anti-SAMHD1 and cells were immediately sorted according to CD45RO and SAMHD-1 expression, using a MoFlo Astrios (Beckman coulter).

### Decross-link and DNA extraction

HIV-1 DNA was purified in column extraction, using the QIAamp DNA Blood Mini Kit (Qiagen) with an additional decross-link step. Sorted cells were washed and resuspended by pulse- vortex in 200 μl PBS 1X, 200 μl lysing buffer AL from the QIAamp DNA Blood Mini Kit supplemented with 20 μl Proteinase K. After 10 min incubation at 56°C, 1.25 mM NaCl was added, and lysates were incubated for another 4 hours at 66°C. 40 μl of Proteinase K was added and lysates were incubated for an additional 60 min at 45°C. Lysates were stored at -80°C or processed immediately.

All reagents used for subsequent DNA extraction were provided from the QIAamp DNA Blood Mini Kit (Qiagen). Following decross-linking steps, tubes were incubated at 100°C for 30 min, mixed by pulse-vortexing every 10 min and cooled at room temperature. Tubes were briefly centrifuged and complemented with 200 μl ethanol (96–100%). Lysates were mixed, applied to the QIAamp Mini spin columns and centrifuged at 6000 x *g* for 3 min. The columns were placed in clean collection tubes after each centrifugation step. Columns were supplied with 600 μl buffer AW1 and centrifuged at 6000 x *g* for 3 min. Another 600 μl buffer AW2 was added and columns were centrifuged at 20,000 x *g* for 3 min, then centrifuged at full speed for 1 min respectively. DNA elution was performed by adding 100 μl buffer AE preheated at 70°C to the columns. After 5 min incubation at room temperature, tubes were centrifuged at 6000 x *g* for 3 min. DNA Eluates of nucleic acid extract were stored at -80°C.

Unfixed cells were used for DNA extraction without decross-link step. 0.5x10^6^ PBMCs samples from each patient were kept for this purpose.

### HIV-1 DNA quantification

Quantification of total HIV-1 DNA was performed using the commercial kit « Generic HIV DNA Cell » (Biocentric, Bandol, France) Viral DNA was quantified in PBMCs and sorted cells by real-time PCR, by detecting and amplifying the LTR region of total cellular HIV-1 DNA, integrated or not. The quantification was carried out on 20 μl of nucleic acid extract, with a maximum of 150,000 cells, according to the manufacturer's protocol. It was calibrated by carrying out and quantifying HIV-1 DNA range of known concentrations (6 to 6000 copies / 20 μl of solution); this DNA was derived from the HIV-1 strain HXB2, produced by the 8E5 cell line. Total cell number of each sample was determined by the quantification of the albumin gene. The amount of HIV-1 DNA copies was expressed per million PBMCs.

### Next generation sequencing and sequence analyses

Deep sequencing of PCR-amplified *env* C2-V3 (HXB2) was performed using the Roche 4.5.4 GS Junior platform (Basel, Switzerland) with microtiter-plate pyrosequencing technology. The different stages of this sequencing required the use of the GS Junior kit emPCR Kit Lib-A (Roche), respecting the manufacturer’s protocol. Read (FASTA) and quality score files produced by the 454 instruments were further analyzed using a bioinformatics pipeline available at https://github.com/veg/HIV-NGS. In brief, high-quality reads were retained and aligned to HXB2 as a reference sequence (without generation of contigs) using an iterative codon-based alignment procedure. Identical sequence reads were clustered, allowing identification of non-redundant sequences. When a cluster contained a minimum of ten identical sequence reads, a haplotype was inferred, and the proportion of reads in each haplotype was collected as previously described [73]. The final output consisted of a list of representative haplotypes and their relative frequencies. For each sample, we then computed the average pairwise distance (APD) between reads with at least 100 overlapping base pairs to quantify nucleotide diversity under the Tamura Nei 93 model [74]. We also quantified the APD (i.e. divergence) between HIV-1 populations originating from different cellular subsets within each host.

Phylogenetic analysis. HIV haplotypes above a minimal frequency threshold of 0.01 were extracted and were used to construct maximum likelihood phylogenies using FastTree [75,76].

### Viral compartmentalization and population structure

Evaluation of the population genetic structure (i.e. compartmentalization) was first assessed by the Fst approach defined as *F*_*XY*_ = 1 - π_I /_ π_δ_, where πI is the estimate of mean pairwise intra-compartment genetic distance (TN93) [77] and πD is its inter-compartment counterpart [78]. Both quantities were computed by comparing all reads from all cellular subsets, subject to the requirement that they share a minimum 150 aligned nucleotide positions. Subsequently, to guard against inference of compartmentalization by skewing of allelic frequencies due to PCR amplification and other biases, we recomputed FST by discarding copy number counts for read clusters (i.e. each cluster was counted as having only one sequence), i.e. all haplotypes are assigned a relative weight of 1. Statistical significance of both tests was derived via 1,000 population-structure randomization/permutation test. We also performed a tree-based Slatkin-Maddison (SM) test for compartmentalization [79]. This method determines the minimum number of migration events between compartments (i.e. cellular subset). Statistical support was determined by comparing this number of migration events to the number of events that would be expected in a randomly structured population [80,81]. HIV-1 sequenced populations were considered compartmentalized if all tests were congruent. Finally, for each discrete ‘location trait’ (i.e. cellular subset compartment), we calculated the association index (AI), Fitch parsimony score (PS), and monophyletic clade size (MC) statistics using BaTS v1.0 [82]. The reported *P*-value is the proportion of trees from the null distribution equal to, or more extreme than, the median posterior estimate of the statistic from the posterior set of trees, and we reject the null hypothesis for a significance level of 0.001, 0.001, and 0.01 for AI, PS, and MC statistics, respectively.

### Statistical analyses

T-test, one-way, two-way analysis of variance (ANOVA) followed by Bonferroni post tests were performed on data in Figs 1, 2, 3 and 5 as mentioned in figures legends. Correlations were evaluated using the Spearman’s rank correlation test. P value less than 0.05 was considered significant. Statistical analyses and graphic representation of the results were performed using Prism (v.5.0b; GraphPad, San Diego, California, USA).

## Supporting information

S1 Fig Represented are mean distances between subsets and mean distances within each subset (Tip size is proportional to distance). Ultra-deep-sequencing (UDS, 454/Roche) of partial *env* (C2/V3) HIV-1 DNA (n = 12 HIV-1 infected individuals receiving c-ART) was performed. Shown are results for the 12 individuals. HIV haplotypes were extracted from reads covering partial *env*; *env* sequences from naive CD4^+^CD45RO^-^SAMHD1^+^, memory CD4^+^CD45RO^+^SAMHD1^+^ and CD4^+^CD45RO^+^SAMHD1^low^ are depicted in red, blue, and green, respectively.
(DOCX)

S2 Fig Slatkin Maddison migration events cross cellular subsets. Viral gene flow across cellular subset using the Slatkin-Maddison (SM) index on phylogenetic trees as implemented in HyPhy. These results suggest limited viral exchange originating from naïve SAMHD1^+^, predominantly directed toward memory SAMHD1^+^ cell subset. Unpaired t test was used for statistical comparison.
(DOCX)

S3 Fig Positive and negative stainings for some of the main monoclonal antibodies used are reperesented. (DOCX)

S4 Fig CCR5 and CD4 expression in CD4^+^ CD45RO^+^ SAMHD1^low^ cells. (a) Gating strategy showing CCR5^+^cells within memory SAMHD1^low^, SAMHD1^+^ and naïve SAMHD1^+^ cells. **(b)** Pourcentages and mean fluorescence intensity (MFI) of CCR5 and CD4 in memory SAMHD1^low^, SAMHD1^+^ and naïve SAMHD1^+^ cells from c-ART HIV-1 infected patients.
(DOCX)

S1 Table Statistical analysis of viral compartmentalization of HIV-1 partial env sequences used in the present study. (DOCX)
