# Supplementary figures and images for: Proliferative memory SAMHD1low CD4+ T cells harbour high levels of HIV-1 with compartmentalized viral populations
Source: PLoS Pathog. 2019 Jun 20;15(6):e1007868. doi: 10.1371/journal.ppat.1007868 (PMC6605680; doi:10.1371/journal.ppat.1007868)

**
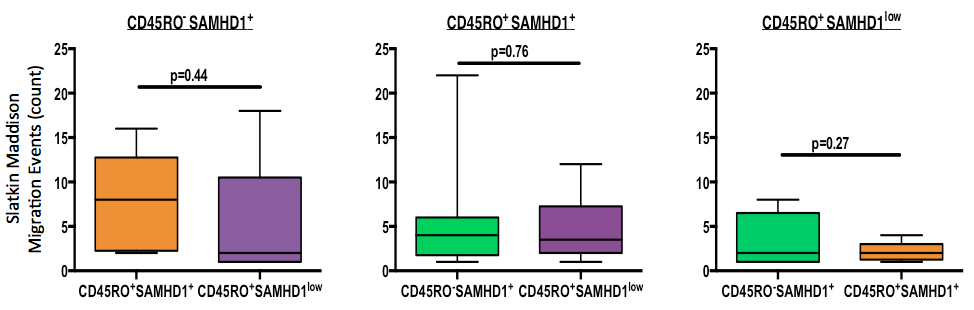
**

**S2 Fig**

Supplement: S2 Fig — Viral gene flow across cellular subset using the Slatkin-Maddison (SM) index on phylogenetic trees as implemented in HyPhy. These results suggest limited viral exchange originating from naïve SAMHD1+, predominantly directed toward memory SAMHD1+ cell subset. Unpaired t test was used for statistical comparison. (DOCX) [file ppat.1007868.s002.docx]

**
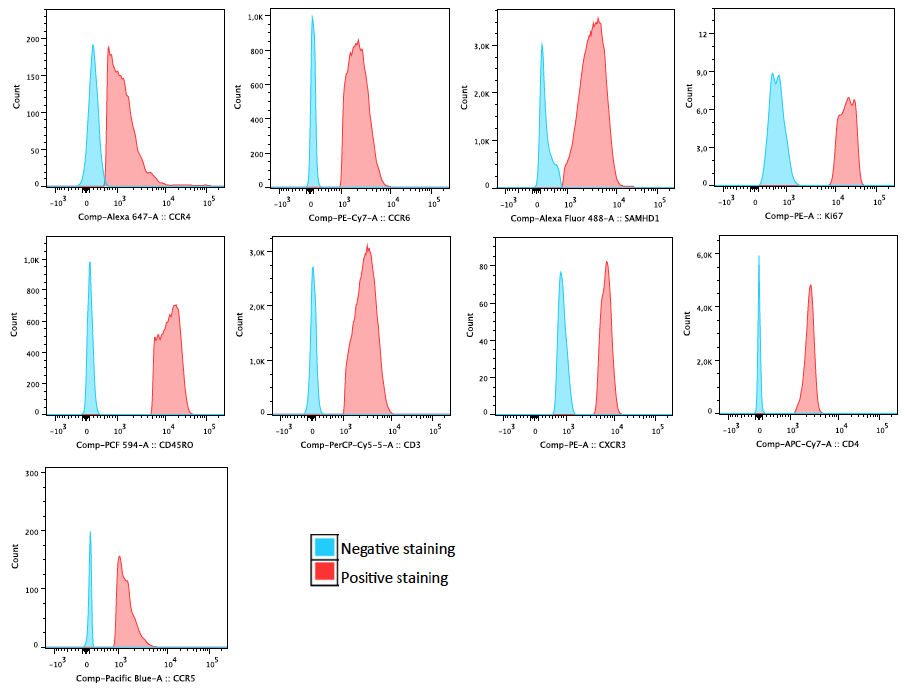
**

**S3 Fig**

Supplement: S3 Fig — (DOCX) [file ppat.1007868.s003.docx]
