# Supplementary material for: Proliferative memory SAMHD1low CD4+ T cells harbour high levels of HIV-1 with compartmentalized viral populations
Source: PLoS Pathog. 2019 Jun 20;15(6):e1007868. doi: 10.1371/journal.ppat.1007868 (PMC6605680; doi:10.1371/journal.ppat.1007868)
Supplement: S1 Table — (DOCX) [file ppat.1007868.s005.docx]

| \| **Individual #11** \|  \|  \|  \|  \| \| --- \| --- \| --- \| --- \| --- \| \| **Statistic** \| **No. of haplotypes** \| **Observed mean (95% CI)** \| **Null mean (95% CI)** \| ***P*-value** \| \| AI \|  \| 3 [2.7-3.2] \| 4.8 [3.9-5.8] \| 0.02 \| \| PS \|  \| 26.8 [26-27] \| 42 [38-45.8] \| 0.04 \| \| MC (CD45RO^-^ SAMHD1^+^) \| 30 \| 4.3 [4-5] \| 2.3 [1.5-3.5] \| 0.05 \| \| MC (CD45RO^+^ SAMHD1^+^) \| 35 \| 5 [4-6] \| 2.7 [2-3.8] \| 0.02 \| \| MC (CD45RO^+^SAMHD1^low^) \| 25 \| 3.3 [2-4] \| 2.1 [1.3-3.3] \| 0.05 \|  \| **Individual #7** \|  \|  \|  \|  \| \| --- \| --- \| --- \| --- \| --- \| \| AI \|  \| 0.2 [0.1-0.3] \| 3.8 [3.1-4.4] \| <0.001 \| \| PS \|  \| 3.8 [3-4] \| 34.5 [30.5-37.5] \| <0.001 \| \| MC (CD45RO^-^ SAMHD1^+^) \| 30 \| 7 [5-12] \| 2.5 [1.5-3.5] \| <0.001 \| \| MC (CD45RO^+^ SAMHD1^+^) \| 24 \| 22.8 [19-24] \| 2.1 [1.5-2.8] \| <0.001 \| \| MC (CD45RO^+^SAMHD1^low^) \| 21 \| 10.8 [2-18] \| 2 [1.3-3] \| <0.001 \|  \| **Individual #12** \|  \|  \|  \|  \| \| --- \| --- \| --- \| --- \| --- \| \| AI \|  \| 2.3 [1.7-2.9] \| 4.5 [3.8-5.3] \| 0.05 \| \| PS \|  \| 19 [17-21] \| 35.7 [32.5-39] \| 0.04 \| \| MC (CD45RO^-^ SAMHD1^+^) \| 28 \| 7 [6-9] \| 2.5 [1.8-3.5] \| 0.05 \| \| MC (CD45RO^+^ SAMHD1^+^) \| 29 \| 3.5 [2-4] \| 2.5 [1.8-4] \| 0.06 \| \| MC (CD45RO^+^SAMHD1^low^) \| 20 \| 5 [4-6] \| 1.9 [1.3-2.5] \| 0.02 \|  \| **Individual #8** \|  \|  \|  \|  \| \| --- \| --- \| --- \| --- \| --- \| \| AI \|  \| 0.5 [0.3-0.6] \| 6.2 [5.3-7.2] \| <0.001 \| \| PS \|  \| 5.3 [5-6] \| 48.8 [44.5-52.5] \| <0.001 \| \| MC (CD45RO^-^ SAMHD1^+^) \| 47 \| 46 [46-46] \| 3 [2-4.3] \| <0.001 \| \| MC (CD45RO^+^ SAMHD1^+^) \| 29 \| 10 [9-11] \| 2.1 [1.5-2.8] \| <0.001 \| \| MC (CD45RO^+^SAMHD1^low^) \| 32 \| 21.3 [14-27] \| 2.2 [1.8-3] \| <0.001 \|  \| **Individual #1** \| \| \| \| \| \| --- \| --- \| --- \| --- \| --- \| \| AI \|  \| 0 [0-0] \| 5.8 [4.9-6.5] \| <0.001 \| \| PS \|  \| 2 [2-2] \| 42.4 [39-45.8] \| <0.001 \| \| MC (CD45RO^-^ SAMHD1^+^) \| 41 \| 23 [23-23] \| 3 [2-4.8] \| <0.001 \| \| MC (CD45RO^+^ SAMHD1^+^) \| 34 \| 34 [34-34] \| 2.6 [1.8-4] \| <0.001 \| \| MC (CD45RO^+^SAMHD1^low^) \| 20 \| 20 [20-20] \| 1.7 [1-2.5] \| <0.001 \|  \| **Individual #6** \| \| \| \| \| \| --- \| --- \| --- \| --- \| --- \| \| AI \|  \| 0 [0-0] \| 2.4 [1.9-3] \| <0.001 \| \| PS \|  \| 2.5 [2-3] \| 19.5 [16.5-21.5] \| <0.001 \| \| MC (CD45RO^-^ SAMHD1^+^) \| 11 \| 4.3 [3-5] \| 1.6 [1-2.3] \| <0.001 \| \| MC (CD45RO^+^ SAMHD1^+^) \| 14 \| 11.5 [9-14] \| 2 [1-3] \| <0.001 \| \| MC (CD45RO^+^SAMHD1^low^) \| 18 \| 11.8 [6-18] \| 2.4 [1.5-3.8] \| <0.001 \| \| **Individual #9** \| \| \| \| \| \| AI \|  \| 0.2 [0-0.5] \| 2.5 [1.8-3.2] \| <0.001 \| \| PS \|  \| 2.5 [1-3] \| 16.7 [14.8-18.5] \| <0.001 \| \| MC (CD45RO^+^ SAMHD1^+^) \| 48 \| 38.8 [38-40] \| 5.7 [3.8-9] \| <0.001 \| \| MC (CD45RO^+^SAMHD1^low^) \| 19 \| 8.5 [3-19] \| 2 [1.3-3] \| <0.001 \|  \| **Individual #5** \| \| \| \| \| \| --- \| --- \| --- \| --- \| --- \| \| AI \|  \| 0 [0-0] \| 6.3 [5.3-7.2] \| <0.001 \| \| PS \|  \| 2 [2-2] \| 45 [42-48.3] \| <0.001 \| \| MC (CD45RO^-^ SAMHD1^+^) \| 28 \| 10.8 [9-13] \| 2.3 [1.5-3.3] \| <0.001 \| \| MC (CD45RO^+^ SAMHD1^+^) \| 40 \| 14 [14-14] \| 3.1 [2.3-4.8] \| <0.001 \| \| MC (CD45RO^+^SAMHD1^low^) \| 30 \| 30 [30-30] \| 2.4 [1.8-3.5] \| <0.001 \|  \| **Individual #4** \| \| \| \| \| \| \| \| --- \| --- \| --- \| --- \| --- \| --- \| --- \| \| AI \|  \| 0.3 [0.1-0.4] \| \| \| 7.4 [6.6-8.3] \| <0.001 \| \| PS \|  \| 5.3 [5-6] \| \| \| 52.6 [49.3-56] \| <0.001 \| \| MC (CD45RO^-^ SAMHD1^+^) \| 41 \| 26.8 [20-31] \| \| \| 2.8 [2-4] \| <0.001 \| \| MC (CD45RO^+^ SAMHD1^+^) \| 33 \| 16.3 [5-26] \| \| \| 2.3 [1.8-3.3] \| <0.001 \| \| MC (CD45RO^+^SAMHD1^low^) \| 38 \| 17.3 [5-31] \| \| \| 2.5 [2-3.3] \| <0.001 \| \| **Individual #3** \| \| \| \| \| \| \| \| AI \|  \| \| 0.9 [0.7-1.1] \| 2.6 [2-3.2] \| \| <0.001 \| \| PS \|  \| \| 6.3 [6-7] \| 19.3 [17.3-21.3] \| \| <0.001 \| \| MC (CD45RO^-^ SAMHD1^+^) \| 13 \| \| 5 [3-8] \| 1.9 [1-2.8] \| \| <0.001 \| \| MC (CD45RO^+^ SAMHD1^+^) \| 11 \| \| 2.8 [2-4] \| 1.6 [1-2.5] \| \| 0.01 \| \| MC (CD45RO^+^SAMHD1^low^) \| 19 \| \| 19 [19-19] \| 2.4 [1.5-3] \| \| <0.001 \| \| **Individual #2** \| \| \| \| \| \| \| \| AI \|  \| 0.8 [0.8-0.9] \| \| \| 3.6 [3-4.3] \| <0.001 \| \| PS \|  \| 5 [5-5] \| \| \| 26.6 [23.8-29] \| <0.001 \| \| MC (CD45RO^-^ SAMHD1^+^) \| 19 \| 5.5 [2-8] \| \| \| 2.1 [1.3-3] \| <0.001 \| \| MC (CD45RO^+^ SAMHD1^+^) \| 28 \| 28 [28-28] \| \| \| 3 [2-4.8] \| <0.001 \| \| MC (CD45RO^+^SAMHD1^low^) \| 14 \| 4 [3-5] \| \| \| 1.6 [1-2.5] \| <0.001 \| \| **Individual #10** \| \| \| \| \| \| \| \| AI \|  \| 1.7 [1.3-2] \| \| \| 2.6 [1.9-3.2] \| <0.001 \| \| PS \|  \| 11 [9-12] \| \| \| 22 [19.5-24] \| <0.001 \| \| MC (CD45RO^-^ SAMHD1^+^) \| 21 \| 7.3 [3-14] \| \| \| 2.4 [1.5-3.3] \| <0.001 \| \| MC (CD45RO^+^ SAMHD1^+^) \| 14 \| 3 [2-5] \| \| \| 1.9 [1-3] \| 0.01 \| \| MC (CD45RO^+^SAMHD1^low^) \| 14 \| 4.5 [4-6] \| \| \| 1.8 [1-2.8] \| <0.001 \| |
| --- | --- | --- | --- | --- | --- | --- | --- | --- | --- | --- | --- | --- | --- | --- | --- | --- | --- | --- | --- | --- | --- | --- | --- | --- | --- | --- | --- | --- | --- | --- | --- | --- | --- | --- | --- | --- | --- | --- | --- | --- | --- | --- | --- | --- | --- | --- | --- | --- | --- | --- | --- | --- | --- | --- | --- | --- | --- | --- | --- | --- | --- | --- | --- | --- | --- | --- | --- | --- | --- | --- | --- | --- | --- | --- | --- | --- | --- | --- | --- | --- | --- | --- | --- | --- | --- | --- | --- | --- | --- | --- | --- | --- | --- | --- | --- | --- | --- | --- | --- | --- | --- | --- | --- | --- | --- | --- | --- | --- | --- | --- | --- | --- | --- | --- | --- | --- | --- | --- | --- | --- | --- | --- | --- | --- | --- | --- | --- | --- | --- | --- | --- | --- | --- | --- | --- | --- | --- | --- | --- | --- | --- | --- | --- | --- | --- | --- | --- | --- | --- | --- | --- | --- | --- | --- | --- | --- | --- | --- | --- | --- | --- | --- | --- | --- | --- | --- | --- | --- | --- | --- | --- | --- | --- | --- | --- | --- | --- | --- | --- | --- | --- | --- | --- | --- | --- | --- | --- | --- | --- | --- | --- | --- | --- | --- | --- | --- | --- | --- | --- | --- | --- | --- | --- | --- | --- | --- | --- | --- | --- | --- | --- | --- | --- | --- | --- | --- | --- | --- | --- | --- | --- | --- | --- | --- | --- | --- | --- | --- | --- | --- | --- | --- | --- | --- | --- | --- | --- | --- | --- | --- | --- | --- | --- | --- | --- | --- | --- | --- | --- | --- | --- | --- | --- | --- | --- | --- | --- | --- | --- | --- | --- | --- | --- | --- | --- | --- | --- | --- | --- | --- | --- | --- | --- | --- | --- | --- | --- | --- | --- | --- | --- | --- | --- | --- | --- | --- | --- | --- | --- | --- | --- | --- | --- | --- | --- | --- | --- | --- | --- | --- | --- | --- | --- | --- | --- | --- | --- | --- | --- | --- | --- | --- | --- | --- | --- | --- | --- | --- | --- | --- | --- | --- | --- | --- | --- | --- | --- | --- | --- | --- | --- | --- | --- | --- | --- | --- | --- | --- | --- | --- | --- | --- | --- | --- | --- | --- | --- | --- | --- | --- | --- | --- | --- | --- | --- | --- | --- | --- | --- | --- | --- | --- | --- | --- | --- | --- | --- | --- | --- | --- | --- | --- | --- | --- | --- | --- | --- | --- | --- | --- | --- | --- | --- | --- | --- | --- | --- | --- | --- | --- | --- | --- | --- | --- | --- | --- | --- | --- | --- | --- | --- | --- | --- | --- | --- | --- | --- | --- |
| AI, association index.  PS, parsimony score. |
| MC, monophyletic clade statistic. |
| 95% CI, 95% credibility interval. |

**S1 Table**
